# Perceptions of and decision making about clinical trials in adolescent and young adults with Cancer: a qualitative analysis

**DOI:** 10.1186/s12885-018-4515-2

**Published:** 2018-06-04

**Authors:** Jennifer A.H. Bell, Victoria Forcina, Laura Mitchell, Seline Tam, Kate Wang, Abha A. Gupta, Jeremy Lewin

**Affiliations:** 1 Joint Center for Bioethics, University of Toronto, Toronto, Canada; 2 Adolescent and Young Adult Program, Princess Margaret Cancer Centre, Toronto, Canada; 3 Division of Medical Oncology and Hematology, Princess Margaret Cancer Centre, University of Toronto, Toronto, Canada; 4 Division of Hematology/Oncology, Hospital for Sick Children, University of Toronto, Toronto, Canada; 5 Department of Psychiatry and Dalla Lana School of Public Health, University of Toronto, Toronto, Canada; 6 Present Address: OnTrac at PeterMac, Victorian Adolescent & Young Adult Cancer Service, Peter MacCallum Cancer Centre, Melbourne, Australia; 7 Division of Hematology/Oncology, Department of Pediatrics, The Hospital for Sick Children, 555 University Ave, Toronto, ON M5G 1X8 Canada

**Keywords:** Adolescent and young adult, Attitudes, Barriers, Cancer, Clinical trial, Beliefs, Psychosocial, Perception

## Abstract

**Background:**

Adolescent and young adults (AYA) enrolment rates into cancer clinical trials (CCT) are the lowest of any age group globally. As AYA have distinct biological, psychosocial and relational needs, we aimed to explore any unique factors influencing their CCT decision-making process, including AYA-specific perceptions or attitudes towards CCT.

**Methods:**

Qualitative interpretive descriptive methodology was used to explore AYA perceptions and decision-making related to CCT. An analytic approach conducive to inductive imagining and exploratory questioning was used in order to generate insights and interpret data.

**Results:**

A total of 21 AYA were interviewed (median age: 31 (18–39)). Twelve (57%) participants had previously been approached to participate in CCT. Major themes influencing trial enrolment decisions were: 1) severity of illness/urgency for new treatment 2) side effect profile of investigational drug in the short and long term (e.g., impact on future quality of life) 3) who approached patient for trial participation (oncologist vs. other) 4) additional information found on-line about the trial and investigators, and 5) family, friends and peer group opinion regarding the CCT.

**Conclusions:**

Several psychosocial and relational factors were identified as influencing AYA CCT decisions, some of which are unique to this demographic. Specific strategies to address barriers to CCT and enable supportive decision-making include: 1) involving family in decision-making and 2) helping AYA appreciate short- and long-term implications of trial participation. Finally, exploring social networking and general education about CCT that AYA can independently access may increase participation.

**Electronic supplementary material:**

The online version of this article (10.1186/s12885-018-4515-2) contains supplementary material, which is available to authorized users.

## Background

Adolescents and young adults (AYA), aged 15 to 39 years, represent a unique subset of cancer patients. With over 70,000 AYAs diagnosed with cancer in the United States annually, and 7600 diagnosed in Canada, this group represents approximately 4–5% of the North American adult cancer population [1–3]. Lack of enrolment of AYA onto cancer clinical trials (CCT) has been the focus of much research [3, 4], predominantly related to investigating system and regulatory barriers impacting access [5]. Examination of perceptions and attitudes towards CCT, also known to impact trial participation, has been limited to children/AYA in pediatric institutes [6–8], older adults [9, 10] and other marginalized populations [11], with few studies focusing specifically on AYA treated in adult cancer centers.

CCT enrolment decisions are influenced by a variety of factors including altruism, scientific benefit, recommendations from medical personnel [12–14] and belief that CCT offers the best treatment [15]. Potential barriers to CCT enrolment include lack of trial awareness [16], concern about side effects [10, 17], medical mistrust, including fear of being a guinea pig [11, 18], protocol stringency [10], belief that risks outweigh benefits [14], concern regarding potential conflicts of interest [19] and lack of trial availability or opportunity to participate [16]. However, there is limited information about the role that personal, socio-demographic characteristics, and other factors might play in the acceptability of CCT for AYA [7, 8, 20–25]. AYA CCT enrolment decisions occur in the context of cognitive and emotional development/growth, and self-identity maturation. As a result, it is likely that unique psychosocial and relational factors place additional stress on and influence their CCT decision-making process [26]. Thus, this study aimed to investigate AYA-specific perceptions or attitudes towards CCT in an adult comprehensive cancer centre and explore additional factors that may influence AYA decision-making about CCT.

## Methods

### Study design

Qualitative interpretive descriptive methodology was used [27]. This discovery-oriented approach allows for a process whereby the researchers remain open to deriving insights from the data in order to inform understandings that answer questions relevant to clinical practice [28]. To do so, in-depth exploration of participants’ experiences is sought with the aim of identifying thematic patterns and commonalities, while simultaneously accounting for individual variation [27]. The overarching goal of interpretive description within this study was to identify clinically relevant insights to support AYA decision making and enhance trial participation. The study was conducted through the AYA Program at Princess Margaret Cancer Centre, Canada [29, 30]. Research ethics board approval was obtained prior to study commencement (CAPCR 16-5376). Participants completed written informed consent generally within 24 h of being approached and prior to any study procedures. They were reminded of their right to withdraw from study participation at any time without affecting their care.

### Sampling and recruitment

Eligibility criteria for participants included: patients aged 15 to 39 years upon cancer diagnosis; currently receiving cancer care (active or in surveillance); and ability to engage in an interview. Those unable to speak English, or who had severe cognitive impairment that would limit their participation were excluded. Patients with leukemia, lymphoma, sarcoma, breast and testes cancer were purposefully recruited with diverse backgrounds with respect to age, clinical trial experience (accept, decline, or neither) and treatment goals (metastatic or disease free).

The principal investigators (AG (female, oncologist); JL (male, oncologist)) who were part of the circle of care identified potential participants during their regular outpatient clinics and made initial contact with them regarding the study. Those patients who were interested in learning more were referred to two members of the study team (VF (female, medical student); ST (female, nursing student)) with whom the patients did not have any previous clinical relationship. These study team members provided a thorough background of the study and patients were given as much time as they needed to consider the study information and provide informed consent or refusal to study participation.

Prior to the interview, participants were asked to complete a demographics form. Participants were given the option to conduct the interview at the current time, or to schedule an interview time in the future. Patients who participated in an interview were given a $10 gift card for their time.

### Data collection

Semi-structured face-to-face interviews were conducted with each participant in a private setting at the hospital. All participants individually engaged in the interview but out of respect for the preferences and desires of some AYA participants, a family member or friend was allowed to be present during the interview to support them. In these cases, the family member or friend quietly observed the interview and only a few interjected occasionally to give additional information that the participant had missed about a certain event or in re-telling a story. Most of the time the family member or friend was silent.

An investigator-developed interview guide was created to explore participants’ understanding of CCT, trial experiences, factors that influence their decision-making about trial enrolment, and AYA-specific perceptions or attitudes towards CCT (see online Additional file 1: Table S1). The guide was influenced by previous literature on the barriers to CCT as experienced by other populations while allowing for open-ended responses. The research team reviewed the guide to ensure that questions were all-encompassing and contained the necessary probes to help facilitate informative responses. Participants were conveyed a working definition of CCTs as defined by the National Institute of Health [31]. Preliminary interviews were conducted and analyzed to further refine the interview guide in terms of applicability, ease of use, and efficiency. Interviews were conducted with one of two investigators (VF and ST) under the guidance of a member of the research team (JB), who has extensive experience and training in conducting qualitative interviews. Interviews were digitally recorded and transcribed verbatim by the researchers (VF and ST). The duration of the interviews was variable (between 30 min to an hour and a half). Transcripts were not returned to participants for comment.

### Data analysis

Qualitative data analysis exists on a spectrum of data transformation from description to interpretation [32]. Because our research aimed to inform clinical practice change, an analytic approach closer to description was used in order to generate insights and interpretation of the interview data related to the research objective [27]. Two members of the research team (VF, ST) independently reviewed three interview transcripts with the intent of developing “broad-based” codes (as opposed to line-by-line coding) ([27], p., 160). These codes were reflective of those ideas, words or phrases identified as relevant to the research aim. Broad-based or generic codes allowed the researchers to remain open to the utterances and meanings within the data so that they might be further interrogated and allow for interpretive thinking [27]. The researchers then met to compare and contrast codes with a third member of the research team (JB), and to establish inter-coder reliability. Consensus was reached between team members (VF, ST, JB) on the creation of new codes or the collapsing of data into existing codes, and the building of detailed descriptions to form an initial coding framework. The initial coding framework was then applied independently by each researcher (VF, ST, and JB) to four more transcripts. The researchers (VF, ST, and JB) met again to discuss any discrepancies in codes and in applying the initial coding framework. Based on this discussion, the initial coding framework was modified and distributed to the entire research team for review. After review and approval, the coding framework was applied by the researchers (VF, ST) to the remaining transcripts. Codes were continually compared between and among transcripts as the researchers asked questions of the data such as “how important is this experience to AYA decision making?” and “is this code qualitatively different than that code?” [28]. Further, potential relationships between identified codes were explored in order to inform a cohesive and clinically-relevant account of influences on AYA CCT decision making [27].

The generation of major themes was guided by a formal code repetition analysis whereby one member of the research team used standard word processing software (Microsoft Word) to count the number of times each code was identified in the transcripts [33]. This exercise served as a proxy for presumed importance of the experience. Major themes were identified as those codes, ideas or experiences that had the most textual support from multiple transcripts. To promote trustworthiness of the findings [34], major themes were reviewed by the entire research team and compared and contrasted with previous literature on CCT participation in diverse populations as well as the clinical experience of the clinician-investigators. In addition, alternate perspectives related to each theme were purposefully sought in order to understand individual variation and to provide a more comprehensive perspective of the collective experience.

## Results

Twenty-three AYA were approached to participate in the qualitative interviews between June and August, 2016. Of the 23 AYA approached, 21 consented to participate. Eight AYA participant interviews included their family member or friend. Participant characteristics are shown in Table 1. The median age of participants was 31 (18-39). Twelve (57%) participants had been approached to participate in CCT of which 10 (48%) had enrolled. The majority of these participants (*N* = 9; 90%) had enrolled at the time of recurrence or during treatment for metastatic disease.

**Table 1:** Participant Characteristics

Total participants (n, %)		21
Age, Median (Range)		31 (18-39)
Gender	Male	10 (48)
	Female	11 (52)
Cancer Diagnosis	Testes	3 (14)
	Leukemia	3 (14)
	Lymphoma	6 (29)
	Breast	3 (14)
	Sarcoma	6 (29)
Previous Trial Involvement	Approached regarding CCT	12 (57)
	Phase I	2 (10)
	Phase I/II	3 (14)
	Phase II	3 (14)
	Phase III	4 (19)
	Not Approached regarding CCT	9 (43)
	Enrolled on CCT	10 (48)
	Enrolled at time of diagnosis	1 (5)
	Enrolled at time of recurrence or during treatment for metastatic disease.	9 (43)
English as first language	Yes	18 (86)
	No	3 (14)
Student and employment status	Currently Working	6 (29)
	Working prior to diagnosis	13 (62)
	Currently at School	3 (14)
Completed university degree	Yes	14 (67)
	No	7 (33)
Children	Yes	9 (43)
	No	12 (57)
	Mean no. of dependent children	1.3
Relationship Status	Single	4 (19)
	In a Relationship	8 (38)
	Married	9 (43)

### Major themes

**Fig. 1:**
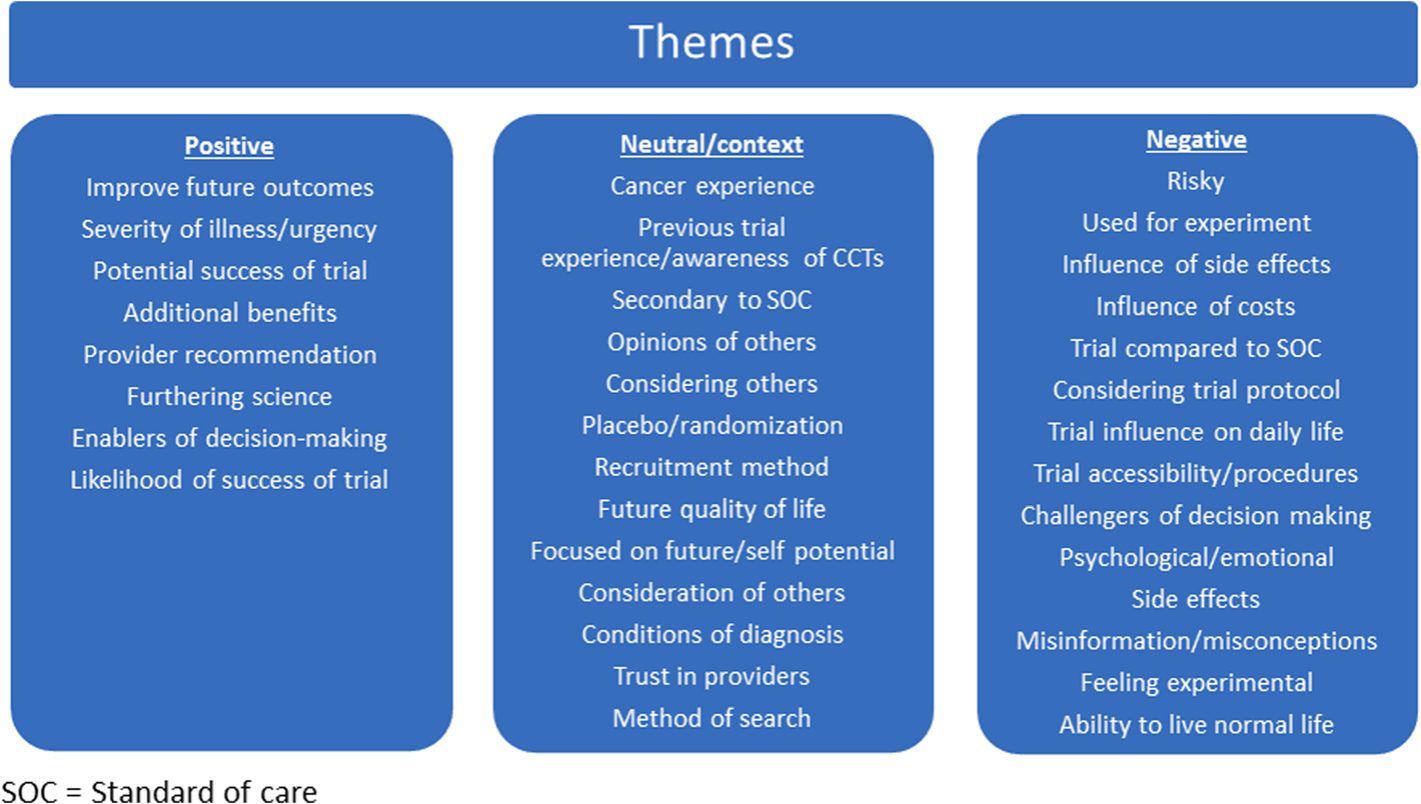
A total of 35 themes were identified as influencing AYA patient decisions regarding enrollment in CCT. These themes were classified as either positive, negative or neutral influencers affecting AYA clinical trial enrollment

Major themes were categorized into positive, negative and neutral influences on CCT participation as guided by the research objective (Fig. 1). Positive influences enabled CCT participation, negative influences detracted from CCT participation, and neutral influences either had no effect on CCT participation or were described by participants as supportive of their trial decision-making process. The top five most frequent thematic influences on AYA decision-making regarding CCT participation, as identified in ≥90% of participant interviews, were: severity of illness/urgency of treatment, influence of side effects, recruitment method, additional information, and opinion of others (Table 2).

**Table 2:** Five prevalent themes influencing AYA decision making were identified in ≥90% of participants

Theme Classification	Theme	Example
Positive	Severity of illness/urgency (95%)	*“I brought up side effects a few times, but if there weren’t really any options or the current options weren’t working and my team was at a loss of what to do. I don’t see that I would have a choice.”*
Negative	Concern about side effects (90%)	*“Yes, it would. Permanent side effects? No thanks. The side effects I’ve already been having with chemo are harsh enough... definitely side effects would be huge thing*”
Neutral	Recruitment method (100%)	*“I was approached very nicely and academically and they gave me the right information. I was able to meet a lot of different professionals working on the trial and felt confident because I got a lot of different perspectives by different teams.”*
	Additional information (95%)	*“I like that I was able to talk about it with my doctor… I know ultimately I make most of the decisions for myself but I hold him in very high regard.”*
	Opinion of others (100%)	*“My friends and family are very informed and aware of what is going on with me. Always want to come to the hospital and they all do their own research…It’s great, I am very supported.”*

#### Positive influences on CCT participation

##### Severity of illness/urgency of treatment

*Severity of illness/urgency of treatment* was discussed by a majority of those interviewed, and was identified by participants as a positive influence on AYA’ willingness to participate in CCT. Most AYA stated they would enrol in a trial out of desperation if they had not responded effectively to the standard treatment available and their disease was progressing or in an advanced stage. As reported by one participant, age 38 (disease free, never been offered a trial), “If I’ve been given a bad prognosis […] I would probably be willing to participate [in a clinical trial], because maybe this new drug might help me”. Similarly, a 36 year-old participant (metastatic disease, currently enrolled on a clinical trial) stated, “I don’t have another option. Radiation is not an option, surgery is not an option, so [a clinical trial] is what I’ve got”. Only one participant said that they would have enough options available so that CCT would not be worth their while. Another participant did not mention severity of illness/urgency as a concern.

#### Negative influences on CCT participation

##### Trial side effects

*Influence of side effects* was discussed in most of the interviews. This theme referred to considerations of how the experience of drug side effects would influence CCT participation. Participants perceived the possibility of trial side effects in diverse ways. It could either act as a deterrent towards enrolling, or it was described as having little to no effect on a patient’s decision to enrol. Over half of participants stated they would consider side effects prior to enrolling in a clinical trial, while only a few participants said side effects were not a major concern. Several participants stated they had no other treatment options available so they would try any treatment regardless of side effects; one participant was concerned only if side effects had a high probability of occurrence. Other participants believed that CCT offered the best chance of cure so they would endure any side effects, or they downplayed the significance of side effects, perceiving they would be easily treated while on trial. Approximately half of participants stated that long-term or severe side effects were quite concerning and that they would need to thoroughly consider their participation prior to enrolment. These participants stated the need to weigh the potential for side effects to impact their future quality of life (QOL) against the therapeutic gains from the trial. One 23 year-old participant (metastatic disease, currently enrolled on a trial) mentioned that, “If [the side effects were] something like numbness [..] I would still go through with [the clinical trial] because my options are getting smaller as more regimens are not working for me. But if it was something pretty serious I would explore my options besides this”. When separated by stage of disease, five of six patients without active disease shared concerns regarding long-term side effects from CCT as opposed to eight of 15 for those with metastatic disease.

#### Neutral influences on CCT participation

##### Recruitment method

*Recruitment method* was described in all of the interviews. This was a general theme used to encompass descriptions of how trial personnel had approached AYA patients about trial participation, and reflections on how this might influence their decision to enrol or not. This theme included participants’ preferred method of being approached about a trial (through email, in-person, or over the phone) along with the timing of approach and from whom they would prefer to hear about a clinical trial for the first time (e.g. from an oncologist, nurse or member of the research team).

More than half of participants said they preferred their oncologist to initiate a trial conversation, while the remaining were ambivalent as long as they had the necessary information to answer any questions they might have about the trial. There was no consensus regarding the preferred timing of being offered a trial or how recruitment should be initiated, although some participants thought they should only be approached about a trial if their standard treatment was not working. Additionally, participants believed an offer of trial participation should not be extended to patients who had just been diagnosed, thinking these patients would be too overwhelmed or not in a stable state of mind to receive further information about clinical trials.

### Additional information

*Seeking additional information* was discussed in most of the interviews. This theme related to the role additional information played in participants’ decisions about CCT participation. Almost all participants reported conducting online research for published studies or gathering more information about the protocol and study investigators. A few participants sought a second opinion from an oncologist who was not their primary physician for more information or to explore additional treatment options. Two participants said they would try to contact previous clinical trial participants to obtain their perspectives and experiences in order to inform their decision making.

### Opinions of others

*Opinions of others* was described in all of the interviews. This theme related to the ways in which the opinions of family members or friends influenced the clinical trial decision. The majority of participants said that family would be involved in the clinical trial decision-making process by helping them to make a decision, supporting their search for more information about the trial, or helping them to stay informed about the clinical trial process throughout recruitment and study participation. Only one participant excluded family in the clinical trial decision. This 28 year-old female explained that she made the decision to enrol in a CCT immediately after the doctor offered the option due to its high success rate. She stated that her only other treatment options would not offer a chance for a full recovery, so the CCT was the best option. Two participants stated that family pressured them with regards to clinical trial participation as opposed to simply offering their opinion. A 21 year-old patient (metastatic disease, had previous clinical trial experience) explained how her father hurried her decision and encouraged her to participate based on altruistic reasons. “[From] my dad, I think it was a bit more of pressure to join the trial […]. If I were alone I would’ve thought about it for a little bit longer, and maybe I wouldn’t have done it”. She stated her father “pushed” her into enrolling in a clinical trial, as it would be “great for research” and “improve the outcome for others.”

## Discussion

As AYA enrolment rates into CCT are the lowest of any age group globally, a major focus of recent research is to understand the barriers to trial participation. In this study of AYA at a large adult comprehensive cancer centre, several overarching themes were identified regarding psychosocial and relational factors influencing AYA CCT decisions, some of which are unique to this demographic.

Many AYA were concerned about both short- and long-term side effects and outcomes of CCT on their future QOL. Of note, we did not specifically probe participants as to whether the toxicity concerns were in addition to standard of care drugs (which are often the comparator arm of many CCT) that often carry their own side effect profile. Although drug side effects are a factor in adult cancer patient trial decisions [10, 35], the priority AYA place on longer-term implications appear to be unique to this cohort. Hope for longevity likely influences AYA’ concerns about the long-term impacts of an investigational agent on future QOL. Whereas older adults may perceive themselves as having lived a significant proportion of their lives, and focus on giving back to others through trial participation [12–24], our findings suggest AYA’ future-oriented considerations may be closely intertwined with stage of disease and considerations of what is best for one’s self in making a trial decision. This emergent finding may have important implications for how trials might generally be promoted or discussed with AYA. For example, public advertisements emphasizing the benefit of helping others may not be as relevant for AYA [36].

Another central theme we identified is method of recruitment. Participants indicated they would not want to be approached for a CCT if they had just been diagnosed or were otherwise overwhelmed with their treatment course. Distress or uncertainty has been identified as a detractor of decision-making ability in a wide range of treatment contexts [37]. Previous literature has demonstrated that adolescents between the ages of 14-18 years may have greater difficulty self-regulating their emotions in response to a life-threatening illness [38], which may make managing their distress in the context of trial decision-making even more challenging. Our findings suggest an important role for professionals to respond to AYA psychological needs, and a CCT recruitment approach that encourages their capacities to make an informed trial decision. For example, approaching AYA about a study using simplified age-appropriate language, acknowledging their emotions and offering further support/resources tailored to their situation, and addressing any practical barriers to CCT participation (e.g., child care, transportation). In this study, AYA preferred their oncologist to introduce them to a trial rather than an independent trial nurse. As such, educating oncologists in adult cancer services of the unique needs of AYAs may aid in recruitment onto CCT.

Finally, family opinion had a profound influence on CCT decision-making. The majority of AYA involved their family in the CCT decision-making process, and perceived their role as supportive in terms of helping them to seek and interpret trial information and make a decision. However, some participants experienced family as an undue influence, particularly when loved ones undermined their autonomy or capacity to fully reflect on their values and determine a course of action.

Shared decision-making is a care delivery model encouraged by the American and Canadian Pediatric Societies [39, 40]. A recent review identified that adolescents and parents prefer partnership and cooperation as opposed to complete independence in decision making about cancer treatments, clinical research, and end-of-life decisions [41]. In fulfilling our ethical and professional obligations, it should not be assumed that every AYA wants to involve their family in decision making to the same extent or in the same way. AYA encompass a wide age range including adolescents aged 15 to mature adults aged 39. For those who are capable, providers should seek direction from AYA about who constitutes ‘family’, and the level of involvement they prefer their family to have [39]. Furthermore, AYA retain the moral and oftentimes legal right to decline CCT participation. In a partnership model, AYA should not be pressured to accept CCT participation against their will or solely on the basis of their parents’ wishes.

A Cochrane review identified interventions to promote participation in shared medical decision making for children with cancer [42]. These interventions included healthcare professional training, and implementation of patient-mediated interventions including decision aids. Although not specifically targeted to AYA, some of these strategies may be tailored for AYA and evaluated within the clinical research setting. Other communication strategies to improve collaboration between physicians, patients and family may also be employed with a goal of evaluating these strategies.

Many AYA used the internet as a primary source for obtaining additional trial information. Current trial databases meant to provide information to patients could be enhanced for AYA [43]. For example, these websites could explore the integration of trial information with online media, social networking sites and online forums as a space for CCT education to enhance AYA knowledge about trials and potentially increase accrual.

We acknowledge several limitations to this study. First, AYA include a wide age range and developmental stages. Given the sample heterogeneity and size, we were unable to explore emergent themes in greater depth, stratified by age, developmental milestones, and clinical trial decision. This is particularly important as 18 was the lowest age range we investigated and one could expect that different themes would be identified in younger versus older AYAs. Not unexpectedly, stage of disease appeared to have interplay with CCT acceptability. As a follow on from this study, we are currently investigating CCT decision making stratified per age group and its interplay with relevant psychosocial, relational influences and stage of disease. In addition, this study was limited to understanding the experiences of AYA who were receiving care at one adult urban comprehensive cancer centre. Future research could involve AYA who are receiving care at community-based cancer settings.

## Conclusion

To our knowledge, this is one of only a few studies examining CCT perceptions of AYA at an adult cancer center aimed at identifying barriers to trial enrolment and enabling supportive decision-making. Several overarching themes regarding important factors and contexts that influence AYA CCT decisions were identified. Specific strategies may include providing opportunity for patients to involve family in decision-making and assisting AYA in appreciating short- and long-term implications of CCT participation. Finally, exploring social networking/online forums and general education about CCT that AYA can independently access, may increase their willingness to participate.

## Additional file

Additional file 1: **Table S1.** Interview guide is presented in the additional file. (DOCX 17 kb)

## Abbreviations

AYA: Adolescent and Young Adult
CCT: Cancer Clinical Trial
QOL: Quality of life
