# Supplementary material for: Perceptions of and decision making about clinical trials in adolescent and young adults with Cancer: a qualitative analysis
Source: BMC Cancer. 2018 Jun 4;18:629. doi: 10.1186/s12885-018-4515-2 (PMC5987432; doi:10.1186/s12885-018-4515-2)
Supplement: Supplementary file 1 — Table S1. Interview guide is presented in the additional file. (DOCX 17 kb) [file 12885_2018_4515_MOESM1_ESM.docx]

**Table S1.** Interview Guide

| 1. Can you give me a quick background of your cancer experience to date, including when you were diagnosed, what treatments you have received, and what treatments you are currently receiving? |
| --- |
| 1. What is your understanding of the purpose of clinical trials?    1. Probe: what do you see as their role in cancer care?    2. Probe: what is your overall opinion of clinical trials? |
| For the purposes of this study, the definition we are using for a clinical trial as defined by the NIH is as follows: *A research study in which one or more human subjects are prospectively assigned to one or more interventions (which may include placebo or other control) to evaluate the effects of those interventions on health-related biomedical or behavioral outcomes.* It would be beneficial if you could use this definition in order to answer the following questions. |
| 1. Have you ever accepted/declined a clinical trial? Can you tell me a little bit about how you first become aware of the clinical trial you are currently part of/ were invited to be part of?    1. Probe: What kind of trial was it? What kind of drug was being tested? What was the study design?    2. Probe: Who approached you?    3. Probe: How was the trial presented to you?    4. Probe: What sorts of information was shared?    5. Probe: Who else was present during the conversation about the trial? |
| 1. Can you elaborate on your experience of making the decision to participate/not participate in a clinical trial?    1. Probe: What sorts of things did you consider?    2. Probe: Did you seek any additional information? E.g., Internet, family, healthcare providers |
| 1. Can you tell me about any conversations you had with family, friends, or other health care professionals about being in a clinical trial?    1. Probe: Were parents/family involved in this decision? If so, how? *(Try to distinguish if family was more pressuring vs. just offering advice)*    2. Probe: Were your health care providers involved in this decision? If so, how? *(Note: C and D will be asked after E if interviewee has not been offered a clinical trial before)*    3. Probe: What was your perception of your doctor’s opinion about being a part of the trial? |
| 1. How informed did you feel in making this decision?    1. Probe: what other information would have been helpful? |
| 1. What factors were/would be especially important to you when making a decision about clinical trial participation?    1. Probe: Side effects?    2. Probe: Travel to the study site?    3. Probe: Social support?    4. Probe: Other life events? (c. and d. only to be asked if previously mentioned by interviewee)    5. Probe: Confidentiality? |
| 1. It is sometimes difficult for researchers to find AYAs for clinical trials. Why do you think that may be the case? |
| 1. What might prevent you from participating in a clinical trial? |
| 1. What may motivate you for participating in a clinical trial?   Probes for I and J:   - 1. Probe: Social support?   2. Probe: Access to drugs?   3. Probe: Urgency of finding an alternate treatment?   4. Probe: Impact of trial on cancer |
| 1. What incentives or disincentives are there for AYAs as a population in general to participate in clinical trials? |
| 1. How do we make clinical trials more accessible to AYA patients? |
| 1. Would you be more likely to enroll on a trial if there was no placebo? For the purposes of this study, we will define the placebo effect as *a beneficial health outcome resulting from a person's anticipation that an intervention—pill, procedure, or injection, for example—will help them, i.e. a physiological response following the administration of a pharmacologically inert “remedy.”* |
| 1. What if there was no randomization of any kind? For the purposes of this study, we will define a randomized clinical trial as *a study in which the participants are assigned by chance to separate groups that compare different treatments; neither the researchers nor the participants can choose which group. Using chance to assign people to groups means that the groups will be similar and that the treatments they receive can be compared objectively. At the time of the trial, it is not known which treatment is best.* |
| 1. Does it depend on who is explaining the trial to you? Doctor vs. nurse vs. someone else? Someone you know vs. a stranger? |
| 1. In your opinion, how could the clinical trial recruitment process be improved? |
